# CRISPR-Based Genome Editing Tools: Insights into Technological Breakthroughs and Future Challenges

**DOI:** 10.3390/genes12060797

**Published:** 2021-05-24

**Authors:** Muntazir Mushtaq, Aejaz Ahmad Dar, Milan Skalicky, Anshika Tyagi, Nancy Bhagat, Umer Basu, Basharat Ahmad Bhat, Abbu Zaid, Sajad Ali, Tanvir-Ul-Hassan Dar, Gyanendra Kumar Rai, Shabir Hussain Wani, Muhammad Habib-Ur-Rahman, Vaclav Hejnak, Pavla Vachova, Marian Brestic, Arzu Çığ, Fatih Çığ, Murat Erman, Ayman EL Sabagh

**Affiliations:** 1 School of Biotechnology, Sher-e-Kashmir University of Agricultural Sciences and Technology of Jammu, Jammu 180009, India; muntazirbt@gmail.com (M.M.); aejazdbt.pbiot@gmail.com (A.A.D.); 2 Department of Botany and Plant Physiology, Faculty of Agrobiology, Food, and Natural Resources, Czech University of Life Sciences Prague, Kamycka 129, 165 00 Prague, Czech Republic; skalicky@af.czu.cz (M.S.); hejnak@af.czu.cz (V.H.); vachovap@af.czu.cz (P.V.); marian.brestic@uniag.sk (M.B.); 3 ICAR-National Institute for Plant Biotechnology, New Delhi 110012, India; tyagi.anshika9@gmail.com; 4 School of Biotechnology, University of Jammu, Jammu 180006, India; nytaneja123@gmail.com; 5 Division of Plant Pathology, Sher-e-Kashmir University of Agricultural Sciences and Technology of Jammu, Jammu 180009, India; basuumar1608@gmail.com; 6 Department of Bioresources, University of Kashmir, Srinagar 190006, India; basharatbhat42@gmail.com; 7 Plant Physiology and Biochemistry Section, Department of Botany Aligarh Muslim University, Aigarh 202002, India; zaidabbu19@gmail.com; 8 Centre of Research for Development, University of Kashmir, Srinagar 190006, India; sajadali84@gmail.com; 9 Department of Biotechnology, BGSB University, Jammu 185234, India; tanvirulhasan@gmail.com; 10 Mountain Research Centre for Field Crops, Khudwani, Sher-e-Kashmir University of Agricultural Sciences and Technology of Kashmir, Jammu 192101, India; 11 Department of Crop Science, Institute of Crop Science and Resource Conservation (INRES), University Bonn, 53115 Bonn, Germany; mhabibur@uni-bonn.de; 12 Department of Plant Physiology, Slovak University of Agriculture, Nitra, Tr. A. Hlinku 2, 949 01 Nitra, Slovakia; 13 Department of Horticulture, Faculty of Agriculture, Siirt University, Siirt 56100, Turkey; arzu@siirt.edu.tr; 14 Department of Field Crops, Faculty of Agriculture, Siirt University, Siirt 56100, Turkey; fatih@siirt.edu.tr (F.Ç.); merman56@hotmail.com (M.E.); 15 Department of Agronomy, Faculty of Agriculture, Kafrelsheikh University, Kafrelsheikh 33516, Egypt

**Keywords:** genome editing, CRISPR, base editing, prime editing, DNA-free genome editing, crop improvement

## Abstract

Genome-editing (GE) is having a tremendous influence around the globe in the life science community. Among its versatile uses, the desired modifications of genes, and more importantly the transgene (DNA)-free approach to develop genetically modified organism (GMO), are of special interest. The recent and rapid developments in genome-editing technology have given rise to hopes to achieve global food security in a sustainable manner. We here discuss recent developments in CRISPR-based genome-editing tools for crop improvement concerning adaptation, opportunities, and challenges. Some of the notable advances highlighted here include the development of transgene (DNA)-free genome plants, the availability of compatible nucleases, and the development of safe and effective CRISPR delivery vehicles for plant genome editing, multi-gene targeting and complex genome editing, base editing and prime editing to achieve more complex genetic engineering. Additionally, new avenues that facilitate fine-tuning plant gene regulation have also been addressed. In spite of the tremendous potential of CRISPR and other gene editing tools, major challenges remain. Some of the challenges are related to the practical advances required for the efficient delivery of CRISPR reagents and for precision genome editing, while others come from government policies and public acceptance. This review will therefore be helpful to gain insights into technological advances, its applications, and future challenges for crop improvement.

## 1. Introduction

Keeping in view climate change, feeding of the global population and addressing the concerns of malnutrition, especially in developing countries from the perspective of global population explosion, have become intimidating tasks for the scientific community [1]. The global population could reach 9.6 billion by 2050, which is an increase from the current 7.3 billion [2,3,4,5]. Conventional breeding approaches appear to be insufficient to fulfill the global food demand and other environmental challenges that we face in the 21st century. To meet the current demand, at least 23 percent more agricultural production is needed. In modern agriculture, breeding through mutations, crosses, and transgenics have become the core crop improvement strategies in the present era. However, conventional breeding being entirely dependent on existing genetic variations and crossing strategies to introduce desirable traits into crops means that it takes a considerable amount of time and resources, which will continue to limit crop improvement. Moreover, genetic variation has significantly decreased, preventing the potential of trait improvements through cross breeding. Mutation breeding has introduced genetic variability through random mutations using physical or chemical mutagens [5,6,7,8,9,10]. Moreover, commercialization of GM/biotech crops is limited by public concerns, as well as by long and costly regulatory evaluation processes [4].

**Figure 1:**
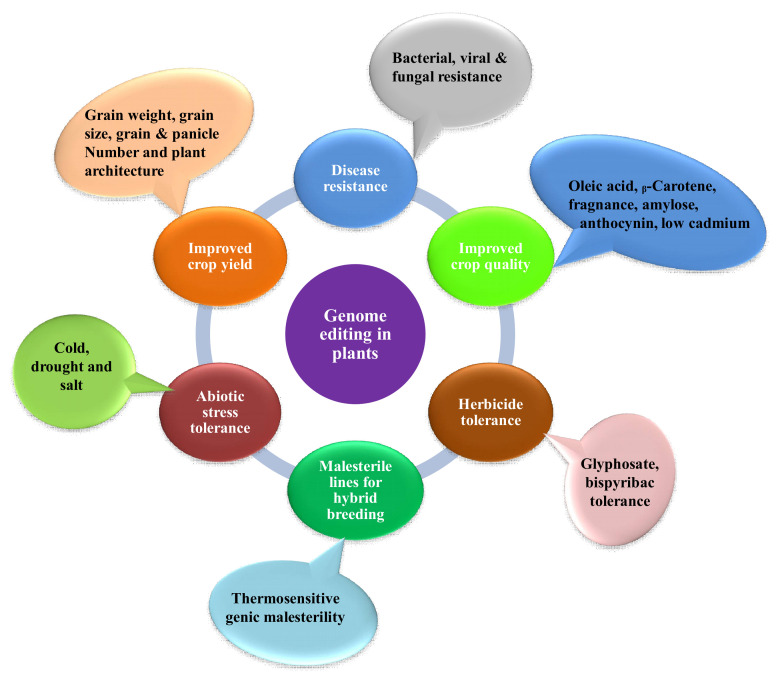
Applications of genome editing in crop plants for improving various traits.

**Figure 2:**
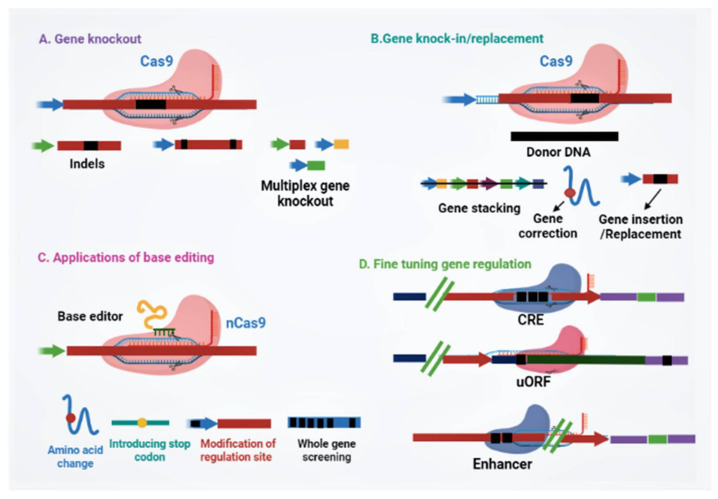
Potential applications of CRISPR/Cas-based applications for plant breeding. (**A**) CRISPR/Cas-mediated mutation can achieve indels, gene deletions, and multiplex gene knockout. (**B**) Gene insertion and replacement mediated by either homology-directed repair or non-homologous end joining can accomplish gene stacking for multiple traits, gene correction for gain-of-function, and gene insertion or replacement to generate novel traits for crop improvement. (**C**) Applications of base editing for crop trait improvement, viz. precise amino acid substitution, gene disruption by introducing a stop codon, gene regulation, and whole-gene screening. (**D**) CRISPR/Cas system-based gene regulation by engineering the regulatory site in the untranslated region, promoter, or enhancer region. Abbreviations: CRE, cis-regulatory element; sgRNA, single guide RNA; uORF, upstream open reading frame.

Since scientists conducted the first gene-targeting (GT) trial in tobacco protoplasts, and the discovery that gene-targeting efficiency is enhanced via DNA double-strand breaks (DSBs) [11], researchers all over the globe have searched for novel developing tools to edit plant genomes. Genome engineering is revolutionizing plant biology through targeted modification of various regulatory elements or genes, or rearranging chromosomes in elite cultivars. Genome-editing employs various sequence-specific nucleases, called engineered nucleases. It consists of two parts: a DNA targeting part that guides the nuclease to the specific target site in the genome and a nuclease that cuts the genome at specific sites. There are three types of engineered nucleases that have been discovered so far: zinc-finger nucleases (ZFN), transcription activator-like effector nucleases (TALEN) and CRISPR-Cas system [12,13,14,15,16,17]. These sequence-specific nucleases generate double-stranded breaks (DSBs) at targeted genomic sites, which are in turn repaired by either homology-directed repair (HDR) or the non-homologous end joining (NHEJ) method [17,18,19,20]. Until now, diverse genome editing methods have been used to improve various traits in crop plants (Figure 1) [18,21]. The CRISPR-Cas9 system, which is being widely adopted for plant genome editing, has accelerated the crop breeding beyond what was imaginable before its development. ZFNs and TALENs suffer from technical complexity and low efficiency, but the CRISPR/Cas9 system is simple and highly efficient. Due to its high efficiency, versatility, minimum cost, and ease of execution, the CRISPR/Cas9 has developed into a potential tool for the improvement of several plant genomes, notably major crop species [5,17,18,22,23]. The application of CRISPR/Cas9 in plant breeding using different editing approaches has been illustrated in Figure 2. A comparative table of three engineered nucleases has been made (Table 1).

## 2. Prokaryotic Origin of CRISPR

CRISPR and its related proteins (cas) play very important roles in providing adaptive immunity in prokaryotes and archaea against viruses and plasmids. It relies on the presence of loci in bacteria, called CRISPR, and it was first discovered in *E. coli*. CRISPR loci consists of operons for the synthesis of the Cas9 protein (nuclease) and repeated spacer sequences. A short fragment (20 bp) of foreign DNA (viral or plasmid) that becomes integrated in bacterial genome is called the protospacer. In the first stage, PAM (protospacer adjacent motif) sequences are identified by bacteria in the invading microbe and then integrates a part of their genome in its CRISPR locus. During the second stage, CRISPR express the entire loci and produces an RNA molecule called crRNA (short for CRISPR-RNA), which, along with Cas9 and other necessary proteins, form a hybrid with invading genomes and Cas9 cuts the genome [29,30,31]. This is how CRISPR works in prokaryotes and helps to protect the bacteria from foreign enemies. This natural system of prokaryotes has been exploited for targeted genome editing in higher organisms, including plants.

A nearly CRIPSR system was developed from the bacterium *Streptococcus pyogenes* (SpCas9), however there are currently many Cas9 proteins known from various bacterial genomes possessing diverse properties [32]. These include a smaller Cas9 derived from *Staphylococcus aureus* (SaCas9) and a Cas9 isolated from *Streptococcus thermophilus* (StCas9), which were discovered to work competently in plants [33]. Besides type II CRISPR/Cas9 systems, type V CRISPR/Cas12a (Cpf1) systems have also been exploited for genome engineering in plants. Type V Cas12a systems are quite dissimilar from Cas9 systems in regards to three features. First, they identify T-rich PAM sequences (TTTN or TTN), which are found just upstream of the non-complementary strand of the target. Second, Cas12a proteins generate DSBs with 5nt 5′ overhangs in place of the blunt ends created by type II CRISPR/Cas9 systems. Third, Cas12a processes its own crRNAs from primary transcripts of CRISPR arrays and there is no requirement of tracrRNAs for crRNA maturation. The exceptional attributes of CRISPR/Cas12a systems label them as an excellent complement to CRISPR/Cas9 systems [34,35]. Additionally, to further expand the genome editing toolbox, engineered Cas9 orthologs with altered PAM sequences and enhanced cleavage specificity have been developed [36]. This includes phage-assisted constant evolution, which has been exploited to produce an ‘evolved’ xCas9 protein that is capable of recognizing a wide array of PAM sequences and reducing the unintended mutations in the human genome. Further, a highly effective SpCas9 variant compatible with ‘NG’ PAM was generated via structure-directed evolution approaches [37], and this Cas9-NG-derived editing system has been demonstrated to be useful in plants [38]. A number of investigations have reported that novel tools that initially evolved for animal cells can also operate efficiently in plant cells [39].

## 3. Working Principle of CRISPR-Cas9 in Plants

**Figure 3:**
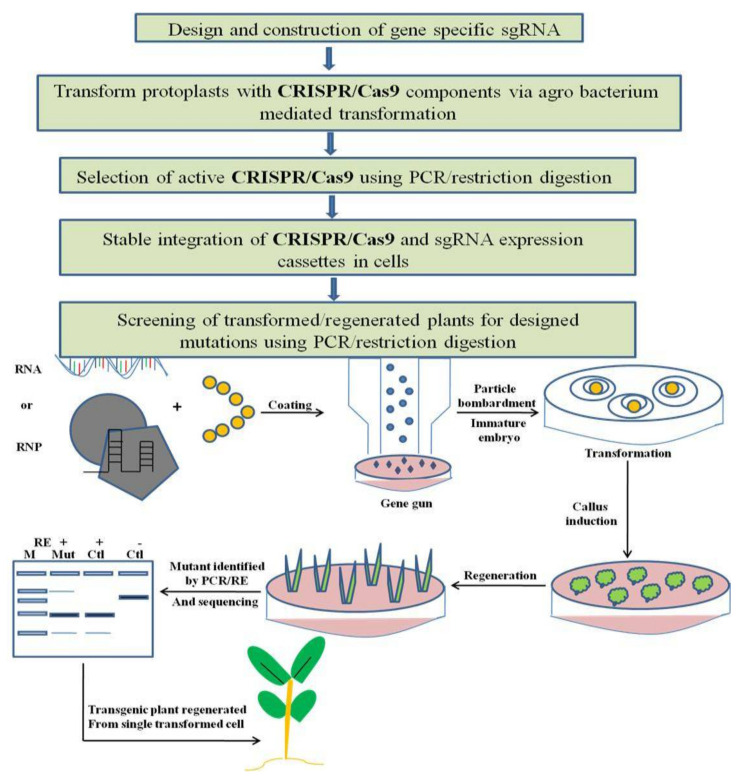
Working principle of CRISPR/Cas9 based genome editing in plants. Plant genome editing can typically be divided into four continuous steps, and the estimated time needed for each step is indicated. PCR/RE, polymerase chain reaction/restriction enzyme digestion. The CRISPR–Cas9 RNAs (in vitro synthesized Cas9 and sgRNA transcripts) or pre-assembled CRISPR–Cas9 RNP can be delivered into immature embryos via particle bombardment. Alternatively, pre-assembled CRISPR–Cas9 RNP can be transfected into plant protoplasts. Bombarded/transfected cells are induced to form calli, from which seedlings are regenerated under the selection-free conditions. Regenerated plants are screened for mutation via the PCR/RE assay and sequencing. Delivering CRISPR–Cas9 reagents via RNP limits their temporal activity, thereby improving their precision. RE, restriction enzyme; M, DNA marker; mut, mutant; ctrl, control.

Generally, CRISPR-Cas-based editing in plant genomes comprises four steps. First, gene-specific sgRNAs (single guide RNA) are designed using different web-based sgRNA design tools, require that users input a genomic location, gene name or a DNA sequence for each gene of concern, and designate a species. The expression of sgRNA in plants is driven by U3 and U6 small nuclear RNA promoters. In order to enhance the accuracy of computational sgRNA selection, organized studies of sgRNA performances in plant cells and large-scale data collection are needed. The second step involves a transient transformation system like hairy-root or protoplast transformation with CRISPR before being used in genome editing, which is the best way to validate the activity of sgRNA. Thereafter, the third step involves delivery of a genome-editing construct into plant cells, usually via particle bombardment or *Agrobacterium*-mediated transformation, and a stable integration of sgRNA and Cas9 into the plant genome. In the last step, screening of transformed or regenerated plants for mutation is done via PCR/RE genotyping and is confirmed by sequencing (Figure 3).

Plant genome-editing with CRISPR/Cas9 raised particular concerns and provided meticulous challenges. One of the major limitations of the CRISPR-Cas9 system is off-target cleavage, which can be deleterious [40]. CRISPR/Cas9 may cleave unintended sequences along with target sequences, since large genomes may contain many sequences of DNA that are identical or similar to target DNA. This may result in unwanted mutations, which may alter other traits [41]. However, various recent studies have demonstrated none to very low off-target editing [42,43]. Moreover, the insertion of CRISPR reagents into plants makes them transgenic and is thus subjected to various bio-safety regulations [17]. The safe and efficient delivery of CRISPR/Cas9 into cells or tissues that are difficult to transfect is another important issue.

In the present review, the current status of plant genome-editing, novel tools and strategies particularly based on CRISPR/Cas9 system and their potential applications in plant biotechnology and agriculture have been apprehensively discussed. We have also highlighted the base-editing tools that allow targeted nucleotide substitutions and describe the DNA-free delivery systems, together with genome editing platforms for crop breeding. Finally, we have discussed the huge potential of genome engineering for plant synthetic biology and challenges and prospects for precision agriculture.

### 3.1. Non-Homologous End Joining (NHEJ) Directed Genome Editing

Modified sequence-specific nucleases are engineered to create DNA DSBs at precise gene loci, facilitating desired genomic modifications via one of the two DNA repair pathways: non-homologous end joining (NHEJ) and homology-directed repair (HDR) [44,45]. NHEJ is the main error-prone repair pathway in response to DSBs as it does not require a homologous template sequence [46] and often creates indels, and can thus create functional gene knockouts [21,47,48]. It can also insert DNA sequences from a donor in a homology-independent manner, and could thus be a proficient strategy for gene integration into a pre-determined locus for crop improvement. NHEJ is evolutionarily conserved and active during the cell cycle, but is the most important during G1 cell phase in which there is no homologous template available for recombination. NHEJ is faster than HDR and repairs most types of breaks in just ten minutes. NHEJ is thus the principal repair pathway that repairs the breaks generated via CRISPR/Cas9 mutagenesis. NHEJ can also be used to produce donor DNA sequence insertions in a homology-independent manner [4] and can thus be an efficient method for gene stacking to improve crops [4]. Sequence replacement or sequence changes can be achieved in plant genomes by utilizing NHEJ.

### 3.2. Homology-Directed Repair (HDR) Mediated Precision Genome Editing

While NHEJ is highly competent and efficient for large-scale gene knockout experiments, HDR-dependent targeted gene editing is still an unprecedented tool for accurate genomic modification and was extensively used in yeast and higher living organisms such as *Drosophila* and mice. Homology-directed repair makes sequence changes or sequence replacements with precision, facilitating precise gene editing (gene targeting). In the case of HDR, the broken DNA ends are repaired on the basis of sequence homology regions from the genome [49,50]. HDR, in principle, may generate gain-of-function point mutations, which can play a crucial role in genetic studies and reveals the gene function. HDR is a small DSB repair pathway in somatic cells that function mostly during the S and G_2_ phases of the cell cycle [48,51]. Even though HR-mediated CRISPR-Cas9 editing in rice and maize has been successful, HDR has been more challenging in higher plants, preventing its widespread application, since target DSB and a repair template must co-exist. Different strategies are used to improve the efficiency of HDR-assisted GT in plants. HDR-mediated genome editing via CRISPR/Cas9 has been successful in various model systems, including human stem cells. Various recent publications report HDR-mediated gene editing using sequence-specific nucleases in *Arabidopsis* [39,52,53], tobacco [39,40,52], tomato [49,51,52], rice [39,52,54,55,56], maize [57], soybean [58], wheat [59], potato [60], barley [61], cotton [62,63] and flax [52,64]. Most of these GT events were based on selection markers, such as herbicide or antibiotic resistant genes at the targeted sites to increase targeting efficiency. However, GT events did not depend on selection markers and showed low frequencies [58,65], hence limiting the importance of these methods. This highly efficient positive-negative selection method has been successful in CRISPR/Cas9 mediated GT in rice. However, this strategy is cumbersome and has been limited to a few resistance genes [4,66]. Moreover, it has not been successful in other plant species, such as tomato and *Arabidopsis* [4,52]. In order to achieve error prone HDR, it has to be restricted to the identical locus of a sister chromatid, and a DNA repair template needs to be delivered into a plant cell. Furthermore, HDR efficiency could be greatly affected by the amount of DNA delivered into a cell. This strategy of increasing the quantity of the donor template in single cells can certainly improve the HDR-assisted GT [4,59,67], which may result in negligible cytotoxicity. Furthermore, the use of a chimeric sgRNA molecule, carrying both sequences for target site specificity and repair template sequences flanked by regions of homology to the target, was demonstrated in a study reporting crop trait improvement in HDR-mediated gene targeting [4,68] and can be used to generate precise gene editing with reasonable efficiency in rice protoplasts.

### 3.3. Beyond Double-Strand Breaks: Recruiting Proteins via dCas9

The HR and NHEJ are the two different repair mechanisms that may be initiated upon DSB detection. However, the CRISPR/Cas9 system is not restricted to generating DSBs, but dCas9 can also offer an exceptional platform for recruiting proteins to regulate gene expression, genome imaging, and epigenomic editing. Fusing dCas9 with a transcription activator or repressor can enhance the transcriptional modulation of targeted endogenous genes in plants. The dCas9 protein is brought to a specific target sequence by a gRNA, thus providing an unparalleled tool to modify genomic sequences at the target sites [69]. dCas9 is now being used in functional genomics to disrupt the gene function through the CRISPR interference (CRISPRi) [70]. The CRISPRi is a highly specific genetic perturbation tool used for transcriptional activation and epigenetic changes with minimum off-target effects. The main advantage of using the CRISPRi tool in plant genome-editing is that multiple sgRNAs can target multiple and different genes simultaneously, therefore enhancing the specificity and efficiency of targeting genes at the same locus [71]. Recently, simultaneous multigene repression and activation was assessed in plants by designing a synthetic repressor system (pCo-dCas9-3X-SRDX) and tested on *CLEAVAGE STIMULATING FACTOR64* gene in *Arabidopsis* and redundant microRNAs: miR159A and miR159B (non-protein-coding genes) [72]. Epigenetic effectors can be recruited by dCas9 to modify epigenetic marks at the DNA or histone targets. Fusion of dCas9 to epigenetic modifiers could help to describe the purpose of methylation and other chromatin modifications responsible for abiotic stress responses in plants [70].

Moreover, fusion of dCas9 and fluorescent proteins, such as GFP, can facilitate visualizing DNA loci bearing repetitive genomic sequences and to label centromeres, pericentric sections, and telomeres using single or multiplex gRNAs [73]. Researchers observed telomere repeats in leaf cells of *N. benthamiana* and observed DNA-protein interactions in vivo by using dCas9 fused with eGFP/mRuby. Therefore, this form of chromatin imaging can be used to study aspects of plant genome architecture [74]. The binding of dCas9 on highly transcribed genes is an active process that depends mainly on transcription activity. Furthermore, dCas9 can access both nucleosome and highly condensed chromatin compartments. Therefore, CRISPR/dCas9 is an efficient tool to employ different fluorescent molecules and effectors to define genomic regions where it can genetically and epigenetically modulate the markers or track the chromatin dynamics in living cells [75].

### 3.4. Fine-Tuning Plant Gene Expression Regulation

In addition to generating mutations in coding regions, gene expression modulation is a constructive approach towards investigating gene functions. Investigations on intracellular pathogen, *Francisella novicida,* led to the improvement of CRISPR/Cas9 as a gene regulatory machinery. The virulence factor that inhibits the bacterial lipoprotein (BLP) production is governed by the *FTN-0757* gene in *Francisella novicida*.*FTN-0757* can also function as a type II Cas9 protein that, in connection with tracrRNA, inactivates the expression of BLP. Precise gene expression modification by means of activation or repression can explain the role of individual genes in many developmental activities [76,77]. Unlike TAL proteins, CRISPR-Cas9 is more suitable for transcriptional regulation because of simplistic engineering. Unprecedented opportunities are presented by dCas9-mediated gene regulation through multiplex gene repression, activation, and epigenome editing to design artificial transcription factors that could be useful to build efficient, complex, and programmable gene circuits. In *Arabidopsis*, a study reported a 400-fold increase in *AtFIS2* gene expression when targeted by dCas9-VP64 to a highly methylated promoter region. The results confirmed that methylated DNA is challenging to target with TAL proteins, but easily accessible by CRISPR-Cas9. Further improvement of CRISPRdCas9 based transcriptional regulation for achieving higher efficiency rates in plants is predicted [78]. In a recent study, promoter regions of genes coding for quantitative traits, such as *SlS*, *SlCLV3* and *SlSP,* were edited for the first time ever, leading to a range of variations and mutated alleles with improved yields in tomato [79].

At the translational level, gene regulation by upstream open reading frames (uORFs) is considered to be a common process that directs protein synthesis from downstream primary ORFs (pORFs) [80]. Researchers reported that the gene editing of uORFs allows for the translational control of mRNAs from four primary ORFs (pORFs) that play a role either in development or antioxidant biosynthesis. They revealed that CRISPR/Cas9 mediated genome editing of endogenous uORFs of *LsGGP2* in lettuceencoded an important enzyme for vitamin C biosynthesis. This editing resulted in enhanced ascorbate content by 80–180 percent and also increased oxidation stress tolerance. This study indicates that editing plant uORFs offers the potential for fine-tuned mRNA translation that could be useful in dissecting biological processes and enhance crop improvement [80].

### 3.5. Engineering High-Efficiency Delivery Platforms for Plant Genome Editing

High efficiency gene-editing entails the delivery of Cas9 protein and gRNAs to target cells. Thus, delivery of CRISPR reagents and genome editing specificity are popular research areas for emerging high-efficiency genome-engineering technologies [21,81]. Plant genome editing technology depends on *Agrobacterium tumefaciens* or direct gene transfer by using biolistic bombardment of gold particles with the help of tissue culture techniques [82,83,84]. Susceptibility of plant cells to *Agrobacterium* infection is a complex trait, resulting in highly diverse delivery efficiencies between species and ecotypes [84]. Microparticle bombardment facilitates delivery of RNA, DNA or protein bound usually to gold particles to the cells. Both particle bombardment and *Agrobacterium* mediated method are generally used to deliver reagents to somatic cotyledonary leaves or true leaves. Despite the fact that both methods are sufficient for transient assays, delivery to non-germinal tissues require further steps to create germinal whole modified plants from the somatic tissue [85]. However, in many crop species, genetic transformation and regeneration from cultured cells is inefficient as it is time consuming in terms of selection and characterization of mutants and can result in somaclonal variations, thus creating additional mutations in their genome and epigenome. Therefore, to quell this problem, using tissue culture-free GE systems, such as viral delivery, ribonucleoproteins (RNPs) and nanoparticle systems offer other alternatives in the GE process. The vehicles that transport GE system cargo are classified into physical delivery [86,87], viral vectors [88], and non-viral vectors [89]. Tissue culture-free plant genome engineering is possibly easier, economical, and less labor-intensive. Simultaneously, it could enhance the efficiency of CRISPR/Cas and decrease the period of generation of edited plants [89,90].

Somatic reprogramming is an evolving alternate to conventional regeneration, in which cell fate is decided by expressing morphogenic genes [91]. Somatic embryogenesis has been accomplished by the overexpression of maize *WUSCHEL* (*WUS*) and *BABYBOOM* (*BBM*) genes. Since being first demonstrated in maize, the use of *WUS* and *BBM* for generating somatic embryos, which develops into a whole plant through many developmental stages, has been revealed to be exceptionally effective and broadly applicable.

Plant virus vectors have rapidly become one of the widespread means for different applications of genome editing, including the commercial production of valuable proteins and gene silencing [92,93]. The efficient machinery and complete genome structure make viruses exceptional vectors [93]. Recently, virus-based vectors with autonomously replicating machinery have been confirmed as an efficient way of delivering GE reagents in plant cells. Both RNA virus (*Tobacco rattle virus*) and DNA viruses (*Wheat dwarf virus*, *Bean yellow dwarf virus* and *Cabbage leaf curl virus*) are efficient gene targets in model plants like *Nicotiana benthamiana* and other important crops (tomato, potato, wheat and rice) [93]. Gemini viruses are plant viruses possessing small circular single stranded (ss) DNA genomes that can efficiently control plant cell functions, entailing immense potential for biotechnology and reverse genetic approaches. Interestingly, various research groups have explained that geminiviral infection promotes somatic homologous recombination and it can be deduced from transcriptome analysis of geminivirus-infected plants [92], though the viral elements necessary for this purpose are left undetermined.

DNA-based delivery of CRISPR reagents has been extensively used in a variety of plant species. However, the protein-based delivery of the in vitro translated Cas9/gRNA RNP complex is still in progress. Delivery of the proteins has some advantages, such as DNA-free delivery, non-transgenic genome edited plants, convenient, cost effective, relatively easy to adapt to high-throughput methods, and low off-target activity. Researchers reported a unique lipofection-mediated gene editing method in plants for the delivery of proteins using preassembled transgene-free Cas9/gRNA RNP [94]. Two lipofection reagents, RNAiMAX and Lipofectamine 3000, were utilized for transfection of non-transgenic tobacco plant protoplasts with Cas9/gRNA RNPs. The maximum efficiencies for RNAiMAX and Lipofectamine 3000 mediated protein delivery were 48 percent and 66 percent, respectively. Additionally, a biolistic technique was established for Cas9-GFP/gRNA RNP delivery into non-transgenic tobacco BY2 lines that rely on the known proteolistic method. These results showed that the Cas9-GFP/gRNA RNP complex maintained integrity while targeting BY2 cells and the intact RNP molecules were effectively transported into the BY2 nuclei. Therefore, the lipofection approach would be a competent method to transport the Cas9-GFP/gRNA RNP into BY2 cells with higher targeted mutagenesis frequency, as compared to biolistic-mediated protein [95] and the PEG-mediated DNA delivery method [96]. Thus, this study finally demonstrated that this novel lipofection-mediated transfection strategy is fit for DNA-free Cas9/gRNA delivery for genome engineering in plant cells. It may possibly expand the use of genome editing to engineer recalcitrant crops and cultivars, as the potential for regenerating a completely new plant with a manipulated genotype from the edited protoplasts [97,98].Besides, gene-edited plants generated via the lipofection approach are transgene-free owing to its transient, DNA-free nature, which is mainly significant for clonally propagated crops, such as sweet potato, potato and strawberry, where crossing and segregation for transgene removal result in a complete change of the genotype [94].

## 4. Availability of Compatible Nucleases

Indeed, numerous strategies and tools have been developed to enhance the target specificity of Cas9, including high-fidelity of Cas9 variants and the Cas9 paired nickase strategy [99]. *Streptococcus pyrogens* SpCas9 is the most common Cas9 used in plants, which recognize the PAM type NGG. While this PAM sequence is widespread across the genomes of plants, it does not cover the whole plant genome. Another problem with SpCas9 is its large protein size (1368 amino acids), which interferes with its delivery through a size limited viral vector, such as the adeno-associated virus (AAV) [100]. PAM sequence requirement restricts the probable target sequences in a gene of interest when it comes to inactivate a gene at any position by targeted mutagenesis. With further studies, the diversity of PAM sequences has been expanded by the novel Cas9 variants and RNA-guided nucleases. Two SpCas9 variants, such as StCas9 [33] from *Streptoccocus thermophilus* and SaCas9 [33,101] from *Staphylococcus aureus,* were found to induce targeted plant genome-editing. They might increase the specificity of editing, as both Cas9 variants require longer PAMs.

Furthermore, seven novels programmable CRISPR-Cas nucleases of the Class 2 system were uncovered in bacterial genomes using functional and computational analyses. These comprise five different DNA targeting nucleases (C2c1, C2c3, Cpf1, CasX and CasY) and two other RNA targeting nucleases (C2c2 and C6c6) that belong to type V and VΙ. The role of Cas12a and Cas12b (Cpf1 and C2c1), Cas12c and Cas12d (CasX and CasY), and cas13a (C2c2) in genome engineering was performed in vitro and/or in vivo by various researchers [102]. Differences between these nucleases in terms of nuclease domains, requirement of tracrRNA and crRNA and the DNA cleavage mechanism, were revealed through functional and mechanistic studies [32].

Cas12a, formerly Cpf1 from *Prevotella* and *Francisella,* is one of the recently discovered and characterized endonucleases from class II type-V CRISPR-Cas systems, which emerged as the most extensively explored substitute to SpCas9 for plant genome-editing [100]. Cpf1 is capable of non-specific cleavage of ssDNA (single-stranded DNA) and RNA, respectively. Cpf1 with the PAM sequence TTTV where “V” is A, C, or G generates staggered cuts with 5′ overhangs [34,99] in a non-specific manner, which is a completely different way from Cas9 that cleaves dsDNA creating, blunt end DSBs. The former cleavage structure offers certain benefits for directed gene insertion utilizing NHEJ [103]. Cpf1 usually generates larger indels (≥6 nucleotides) besides possessing not only DSB-inducing activity, but also RNase ΙΙΙ activity implicated in pre-crRNA processing [100]. This activity of Cas12a can be exploited in multiplex genome-editing through a tandemly arrayed pre-crRNA-expressing construct [102]. Genome-editing via Cpf1 has been already tested in various plants. Surprisingly, highlighting the high specificity of Cpf1, there was no off-target edits found in these plants [99]. Such novel CRISPR-Cas tools offer potential applications, as well as to tackle specific challenges facing plant genome editing. A group of researchers demonstrated that Cas12a is involved in transcriptional repression in plants; hence, this system, besides being used in genome-editing, can act as an attractive platform for regulating gene expression in plants [104]. Cpf1 may also cleave RNA, which further enhances the functionality to this class of nucleases [105]. Moreover, the capacity of multiplex genome-editing can be improved by the combination of different CRISPR systems [106].

Agricultural production is severely affected by RNA viruses, in contrast to DNA viruses. Cas13a is currently advancing plant biology research and is a promising toolbox in the CRISPR arsenal to contribute to global food protection and do not depend on transgenes. It has been used to combat tobacco RNA viruses and offers a novel technique to confer immunity [107].

## 5. Breakthrough Technologies in Generating DNA-Free and Genetically Stable CRISPR-Edited Plants

**Figure 4:**
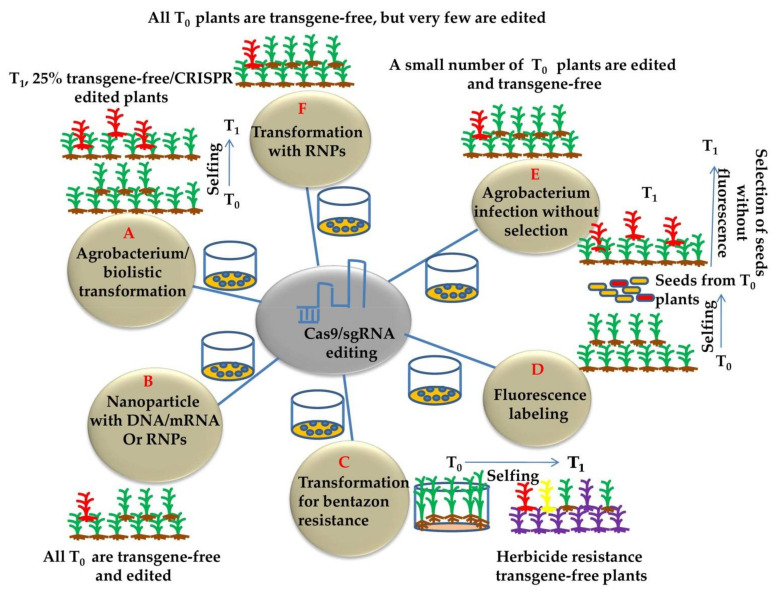
Commonly used approaches for developing transgene-free and CRISPR-edited plants (**A**) CRISPR gene editing followed by agrobacterium/biolistic transformation obtaining 25% T_0_ transgene free plants by following Mendelian segregation. (**B**) Nanoparticle and RNP mediating gene editing. DNA, RNA, or RNP coated nanoparticles can deliver CRIPSR reagents into meristematic cells. This strategy typically produces mosaic plants. The transgene-free and edited plants may be obtained by either sexual or asexual propagation from the edited tissues. (**C**) Drug-induced elimination of transgenes. The CYP81A6 encodes an enzyme that metabolizes bentazon, which is a herbicide. Coupling CYP81A6 RNAi with CRISPR components enables a selection for transgene-free and edited plants. (**D**) Fluorescence labeling and selection of transgene free plants. The mCherry fluorescence marker is linked to the gene-editing components in the same plasmid. The marker allows for the selection of transgene-free seeds, greatly reducing the workload associated with growing plants and genotyping. (**E**) Generation of edited plants using transient expression under no selection pressure. In the absence of a selection pressure, Agrobacterium infection can lead to the expression of transgenes without integrating the transgenes into chromosomes. Such events can lead to the generation of transgene-free and edited plants. (**F**) Ribonucleoprotein or RNP (Nuclease and gRNA) method generating transgene free plants by particle bombardment/gene gun into calli or immature embryos.

Plant genome-editing involves the transformation of plant cells with the CRISPR/Cas9 construct and the regeneration of whole plants from a few transformed cells. Genome editing has generally been applied to transformable plants, and existing genetic transformation systems are usually specific to the genotype and have been developed in many plant species. *Agrobacterium*-mediated plant transformation was used extensively as a flexible tool for generating stably transformed model crop plants [108]. The different methods for generating transgene-free and CRISPR-edited plants are illustrated in (Figure 4). Alternatively, *Agrobacterium* can facilitate transient transformation of plant cells [109,110] and can be used to transiently express Cas9 and gRNA for producing transgene-free genome editing in plants (Figure 4A). This approach has been effectively used to edit the tobacco *phytoene desaturase* (*PDS*) gene using the vector system devoid of a selection marker to permit the survival of transiently transformed cells [111] (Figure 4E). In general, 10% of tobacco plants were transgene-free and edited. Moreover, no sexual segregation is required for the removal of transgenes. It is still difficult to detect the desired transgene-free plants using this method. Using *Agrobacteria* engineered to enhance the production of DNA-free gene edited plants and/or to enhance the transient expression of transgene in plant cells will significantly enhance the efficiency of transgene-free edited plants. Moreover, *Agrobacterium* is used to transform most plants, but being a plant pathogen raised some regulatory concerns. The potential of plant genome engineering lies within the successful plant transformation. Delivery of CRISPR/Cas9 reagents and plant transformation problems are the major issues of concern. Moreover, the focus should be on novel tools and strategies that unify the approaches across plant species [99].

Most plant genome-editing approaches involve the delivery of the expression cassette CRISPR-Cas9 into plant cells, its integration and expression into the nuclear genome and finally the cleavage of the desired nucleotide sequence. However, a small part of dispatched DNA sequence becomes incorporated into the genome. It has also been observed that CRISPR/Cas9 can be transiently expressed and may provide an alternative to plant engineering. Recently, wheat has been transformed via two easy and proficient genome-editing methods that depend on the transient expression of CRISPR-Cas9 RNA or DNA [112]. These two methods removed the selection stage for antibiotics or herbicides in tissue culture during post-transformation stage and callus cells transiently expressing CRISPR-Cas9 were used to regenerate plants. Consequently, tissue culture techniques used were time efficient and less labor intensive. Moreover, transient gene expression systems significantly reduced the transgene integration in wheat callus cells [112]. Genome-editing via CRISPR-Cas9 DNA or RNA transient expression has been established in callus cells of wheat, and can also be successful in other plant species.

Plants stably transformed with CRISPR-Cas9 by harboring insertions and deletions (indels) at the target loci may often contain undesirable mutations at both on-target and off-target sites [113]. The use of gene-editing in crop biotechnology has been limited, as genome-edited plants were often considered to be genetically modified organisms (GMOs) and strongly regulated in many countries. Since genetic segregation can remove the foreign gene, but in asexually reproducible plants, this is not feasible. Moreover, excision of the CRISPR/Cas9 transgenes by genetic segregation and backcrossing is time consuming and arduous [114]. In addition to being foreign to DNA, gene-edited plants have not been approved by some regulators since recombinant DNA constructs were used in their development [115]. Therefore, efforts have been made by various research groups throughout the world in developing methods to generate CRISPR-edited DNA (transgene)-free plants. Advances in DNA-free genome editing involve the delivery of a mix of mRNA and gRNA encoding Cas9 [112] or pre-assembled ribonucleoproteins (RNPs) (Figure 4F) [116]. The RNPs have turned out to be a favorable tool for DNA-free genome-editing in crop plants. Recently, protoplasts of four plant species transfected with RNPs induced successful targeted gene modifications [117], but only mutant lettuce plants from RNP-transfected protoplasts may be regenerated [117]. At present, a small number of plant species, few of which are monocots, are being regenerated from protoplasts. Alternatively, particle bombardment was used to deliver RNPs into maize and wheat embryos to harvest gene-edited plants [57]. Genotype independent approach in the study confirmed that overexpression of Wuschel2 (*Wus2*) and Baby boom (*Bbm*) from maize (*Zea mays*) improved transformation frequencies in maize, rice (*Oryza sativa* spp. *indica*), sugarcane (*Saccharum officinarum*) and sorghum (*Sorghum bicolor*) [118]. Toda and coworkers demonstrated the DNA-free gene-editing method through direct delivery of Cas9-gRNA RNPs, which were transfected into rice zygotes developed by in vitro fertilization of isolated gametes [119]. The mature plants grown from zygotes without the use of selection agents resulted in regenerating rice plants with targeted mutations in 14–64% of plants. This proficient DNA and selectable marker-free genome editing approach has tremendous potential for improving rice and other crop species of economic importance. Moreover, various recent studies have reported DNA-free CRISPR/Cas mediated plant transformation (Table 2). This approach should be optimized, along with the identification of more *Wus2*- and *Bbm*-like genes, to attain effective plant transformation and to increase the scope plant genome editing.

Recently, a group of researchers developed a method that eliminates plants with CRISPR constructs in a drug-dependent manner by taking advantage of the RNA interference (RNAi) [131]. Rice plants with cytochrome P450 *CYP81A6* gene makes them resistant to sulfonylurea and bentazon herbicides, and thus the plants with *CYP81A6* showed hypersensitivity to bentazon. This strategy can significantly reduce the labor required to select transgene-free plants (Figure 4C).

CRISPR-mediated gene editing depends on the efficient delivery of gRNAs and nucleases in plant cells, but transformation is difficult in many plant species. Nanoparticles can be used to deliver CRISPR components (either DNA/RNA or RNP) into plant cells beyond transformation (Figure 4B). A variety of nanomaterials, such as magnetic nanoparticles (MNPs), carbon nanotubes (CNTs) and carbon points, were used in plant genetic engineering as delivery vehicles [15,132,133]. Magnetofection was described as a promising and highly efficient method of the gene carrier for plant genome editing. It works on the principle of magnetic force to promote DNA uptake associated with MNPs into targeted plant cells [134,135,136]. Magnetic nanoparticles can link pollen-grains that are made possible by magnetic force. The pollen grains infiltrated by nanoparticles are still viable, remain viable after (gene delivery), can pollinate cotton plant, and can successfully introduce marker genes into cotton, indicating that transformation-independent platforms of genetic engineering could be possible in plants. This method is simple, rapid, culture-free, genotype independent, and capable of multi-gene targeting [125,137]. However, there are no reports of transient transformation via pollen magnetofaction in several monocot species. Vejlupkova and coworkers were unable to reproduce any evidence of transient transformation in lily pollen via magnetofection [138], as reported by Zhao et al. [137]. CNTs can be used to deliver biomolecules in protoplasts and plant cells without degradation by cellular metabolic enzymes [136,139,140]. At the University of California, it has been established that diffusion-based biomolecules, such as DNA or other cargo, could be delivered into intact plant cells in a species-independent approach [136,139,140]. Recently, LEEP (Lipid Exchange Envelope and Penetration) model was exploited to alter nanomaterials for delivery of biological molecules to particular plant organelles [141]. Chitosan complex SWCNTs (CS-SWCNTs) may deliver plasmid DNA selectively to tobacco, watercress, spinach and *Eruca sativa* plants in chloroplasts. In a similar study, arginine functionalized SWCNTs (Arg-SWCNTs) and chimeric peptides were engineered for the delivery of DNA into the intact root cells of tobacco, resulting in plant transformation. Recently CNTs were used for gene transfer in German chamomile cells [142]. However, there is currently no study on CNTs delivering gene-editing components into plant cells for editing target genes successfully [143].

Nanoparticles may supply gene-editing reagents to any plant cell, including meristematic ones, thus offering great potential for plant genetic transformation over conventional methods of gene transfer [136,144]. While the capability of nano-biotechnology in plant gene editing is still in its infancy stage, including the nanoparticles safety for human health and the surrounding environment, huge potential and progress in our current understanding of nanoscience would still accelerate the progress of nanomaterials as an incredible tool for plant genome engineering.

## 6. Methods for Detection of Mutation/Edits Created by CRISPR/Cas9

The most important and challenging step after the successful delivery of CRISPR/cas9 cassette is the (a) selection of transformants with mutations and (b) the elimination of CRISPR–cas9 cassette, as the presence of it in transformations can increase the rate of off targeting [126,145,146]. However, by selfing and back crossing CRISPR-cas9 cassette can be eliminated in T_2_ generation and Cas9 free mutants can be produced. Various methods can be employed for the detection of mutation, which ranges from noticeable phenotypic changes to molecular characterization of transformants.

### 6.1. PCR Based Detection

One of the most common and effective methods for the detection of edited DNA sequences in genomes is PCR. Various PCR-based techniques have been developed to identify homozygous/biallelic mutants. If a sequence of target DNA is known, then complementary oligonucleotide primers can be applied for the amplification of the targeted region. Guo and coworkers developed an efficient and cost effective mutation-site-based specific primers Polymerase chain reaction (MSBSP-PCR) method for identification of homozygous/biallelic mutation in *Nicotiana tabacum* and *Arabidopsis.* Additionally, this method could be utilized in other plant species for the identification of mutants based on its on and off potential [147]. Another group of scientists demonstrated a method that detects mutations on the basis of single strand conformational polymorphism (SSCP) for the successful detection of mutants in rice [148]. This technique involved the PCR amplification of the mutated region using a specific set of primers and denaturation of the amplified product, and the mutated region was identified by SSCP analysis, as the mutated single strand will migrate differently from the wild type on PAGE. By this method, they successfully identified multiple *OsROC5* and *OsDEP1* mutants in rice.

### 6.2. Sequencing Based Detection

Sequencing is a powerful method for the detection of mutation in the genome by sequencing the region that contains mutation. There are different sequencing techniques available. Sanger sequencing (first generation sequencing method) works well if targeted sequence is known [149]. Another way is whole genome sequencing using next generation sequencing (NGS) platform. WSG does not require the prior information of targeted site, as in case of Sanger sequencing. NGS works on the principle of massive parallel sequencing that generates millions of reads at once, but the disadvantage is the size of the reads (300–700 bp), so large indels cannot be detected [149,150]. This limitation of the NGS has been overcome by third generation sequencing methods such as PacBio. PacBio sequencing is powered by Single molecule real time sequencing technology (SMRT), which can sequence an average length of (8.5 kb) and hence large indels can be identified, but SMRT has a higher error rate compared to NGS and Sanger sequencing.

### 6.3. Fluorescence Marker Assisted Detection

Fluorescence markers are the visual markers used in various studies for the selection of predicted mutants based on its on and off potential. In an investigation, the *mCherry* fluorescence marker was linked to CRISPR constructs in the same plasmid in order to use *mCherry* as an alternative for transgenes [145]. Additionally, *mCherry* was controlled by a strong seed promoter *At2S2*, enabling the easy detection of transgenic seeds from non-transgenic seeds, thus significantly reducing the labor required to obtain CRISPR-edited transgene-free plants. The CRISPR-assisted fluorescence marker technology can be used simply in other plants (Figure 4D). This technology has significantly simplified genome editing in *Arabidopsis* [151] and rice [152]. Transgenic T_1_ seeds of edited plants can be easily recognized by the intense red florescence. As Cas9 and gRNA are associated with the same plasmid, the fluorescence intensity generally correlated with their expression levels, thus providing evidence to select plants with high gene-editing efficiency. While this technology reduces labor by 75%, the method still needs a substantial amount of effort and time [153]. In some cases, gene silencing of the fluorescence gene occurs in mutants, so in order to confirm, PCR should be combined with fluorescence marker screening [154].

### 6.4. Transgene Killer CRISPR Technology

It has been demonstrated that Transgene Killer CRISPR technology (TKC technology) improved transgene-free and CRISPR-edited plant recognition by deliberately and automatically deleting any plant comprising the CRISPR/Cas9 structure [155]. They employed a pair of suicide transgenes that successfully killed all CRISPR/Cas9 containing T_0_ generated pollen and embryos, and consequently eliminated all the transgenes effectively from the T_1_ plants. This CRISPR architecture would significantly save the time and energy needed to separate transgenic-free and edited plants. This technology has been successfully used to isolate CRISPR/Cas9 edited and DNA-free rice plants [155]. Researchers selected the *OsLAZY1* gene to validate the efficiency of this technique in isolating transgene-free gene-edited plants [153]. A molecular investigation confirmed that all the progeny obtained from T_0_ plants using TKC technique were transgene-free, while at least 75% of the progeny generated from T_0_ plants from customary CRISPR vectors still had the transgenes. Further, it was demonstrated that the TKC system is very competent in both gene editing and in isolating the transgene-free plants as all the T_1_ plants studied were gene-edited and transgene-free [155]. The TKC method can be easily utilized and TKC vector construction involves fewer steps compared to the traditional CRISPR vectors. Conventional cloning techniques are used to insert specific gRNA cassette into the TKC plasmid vector. Moreover, transgene-free and genome edited plants can be generated in a single generation using TKC-enabled genome engineering. Additionally, to assess the applicability of TKC technology, plasmid TKC-D3 was generated in order to target the gene *DWARF3* (D3) involved in the reaction to strigolactone [155]. Additionally, transgene-free progenies were produced from two separate plants at T_0_ generation [155].

In the beginning, the CaMV35S promoter was used by TKC vectors only to drive the expression of CMS2 killed pollen cells, and thus the presence of ubiquitous CMS2 in plants is not perfect. Various reports revealed that CMS2 protein accumulation increased the susceptibility of plants to drought, as compared to wildlife [156]. Furthermore, promoter *CaMV35S* does not fit in monocots than dicots [157] and often experiences epigenetic changes, which lead to gene silencing [158]. TKC technology has been improved by offering two novel TKC vectors that replace the CaMV35S promoter with two rice promoters in order to drive the suicide genes that accelerated gene editing efficiency and transgene removal from CRISPR/Cas9-edited rice [155]. Novel TKC vectors could offer advantages in contrast to TKC1.0 under certain circumstances. This improved TKC technology offers more opportunities and plasticity to carry out gene editing in rice, which may subsequently speed up crop improvement [153].

In conclusion, the marker-assisted selection of transgenic-free CRISPR-edited plants and TKC technology are highly effective in the elimination of transgenes in sexually reproductive plants. However, the RNP-based DNA-free technology, nano-biotechnology, and short-term expression of CRISPR genes allows for the production of transgene-free edited plants for non-sexually reproduced crops, such as citrus and grapes. To eradicate transgenes efficiently and easily in both non-sexually and sexually crops, further updating of the current technology and the creation of novel technologies are also needed. This strategy can be easily adopted for other plant species, including woody plants that can be transformed through tissue culture and even more beneficial for crops that have long life cycles and produce fewer seeds.

## 7. Multi-Gene Targeting and Complex Genetic Engineering

The CRISPR/Cas system can be used as an efficient and precise gene-editing vehicle for multiplexed genome editing, i.e., the simultaneous targeting of multiple genes with a single molecular construct uses multiple sgRNAs as a separate transcription unit or in polycistronic form. This is one of the major advantages of CRISPR/Cas9 technology, as compared to previous genome editing tools, e.g., ZFNs and TALENs. It is easy and simple to use more gRNAs on the similar or other T-DNA construct. Multiplex genome-editing can be achieved by using multiple promoters, but a single promoter is preferable in order to express each gRNA and to fit the entire system into smaller vectors, provided the effectiveness of the mutations is maintained. This was seen with a polycistronic gene spacing the gRNAs with tRNA genes, Cys4 recognition sites and ribozyme sites [4,105]. CRISPR/Cas9 mediated targeted editing by the ribozyme-gRNA-ribozyme (RGR) system in the case of *Arabidopsis* and yeast has been reported [158]. The RGR system has been used to describe the single promoter-driven CRISPR/Cas9 mediated mutagenesis in mammalian cells [158,159]. Recently, it has been reported that hammerhead ribozyme, one of the RNA processing machineries, could be used to produce functional gRNAs to express CRISPR/Cas system in plants [160]. The Cys4 processing system that utilizes the CRISPR type III RNase, Cys4, to cut the 20 bp sequences flanking the gRNAs can be used for successful CRISPR/Cas9-mediated targeted mutagenesis in plants [67]. Besides, multiplex gRNAs has been generated from a single transcript using the polycistronic tRNA-gRNA genetic system in plants [161]. Various facts suggest that the methods of tRNA and Cys4 using the cestrum yellow leaf curling virus (*CmYLCV*) promoter are unsurpassed in inducing mutations, as compared to the gRNAs expressed from individual Pol promoters [67]. Thus, the efficiency of CRISPR/Cas9 mediated targeted mutagenesis can be improved by Pol II promoters to express gRNAs in comparison to Pol III promoters. Multiplex genome-editing has been achieved in tobacco BY-2 suspension cells and rice protoplasts transcribing gRNAs spaced with tRNAs. Multiplex genome engineering has also enabled large chromosomal deletions utilizing two gRNAs targeting different sites. Moreover, any deleted region can be replaced with a preferred gene by homologous recombination, provided that the gene of interest should be flanked by sequences corresponding to target regions of the gRNAs [162]. Recently, the CRISPR/Cas9 system was used successfully to target several genes in protoplasts and suspension cultures of wheat. Genome editing has been demonstrated using *Agrobacterium*-mediated transformation in bread wheat in PEG-transfected protoplasts, cell suspension cultures and electroporated microspores. Regeneration of edited wheat plants was accomplished by immature embryos, immature embryo-derived callus or apical meristems fired by particle bombardment or agrobacterial transformation. Multiplexed genome targeting in multiple loci has also been performed in polyploidy wheat genome by homologous recombination utilizing virus based CRISPR/Cas machinery. Tobacco rattle virus has been used to transform tobacco plant with RNA to insert multiple gRNAs overexpressing Cas9 resulted in multiplex genome-editing [163]. Thus, multiplex genome-editing by using the CRISPR system in *Arabidopsis*, maize, wheat and tobacco has revealed the significant advantages of RGENs over other programmable nucleases. In addition, Cpf1’s superiority in manipulating its own crRNA provides a competent tool for editing multiplex plant genomes. Thus, the CRISPR platform represents a highly effective gene pyramiding system as it enables simultaneous multiple-trait modifications.

## 8. Base Editing for Plant Breeding and Crop Improvement

Recently, a novel technique based on the CRISPR-Cas9 system termed, ‘base editing’, was developed. It edits single DNA bases at the specific sites in the genome without creating DSBs or incorporating a foreign DNA template and is HDR independent [164]. Base editing could be more efficient in targeting base correction than HDR-mediated gene replacement with low rates of indel formation. Furthermore, it is more difficult to generate an HDR plasmid than to construct a base-editing vector. Since its development in 2016 by David Liu and colleagues [69], various base-editing tools have been developed to insert point mutations in both dividing and non-editing cells of diverse array of prokaryotes, fungi, animals, plants, and microbes. Rapid developments in base editing have considerably reduced undesired editing, thereby increasing the space and efficacy of genome editing. Base editing can install all the four transition mutations, in all the four bases (Guanosine, Adenine, Cytosine and Thymine), G → A, A → G, C → T and T → C in the genome with the existing CRISPR/Cas base editors. The cytosine base editor (CBE) can insert a C– G to T–A mutation, whereas the adenine base editor (ABE) can change an A–T base pair into a G–C pair. In RNA, Adenine (A) to Inosine (I) conversion is also possible with the aid of the RNA base editor (RBE) [164]. The applications of CRISPR/Cas-mediated base editing in plants has been highlighted in Table 3.

In a recent study, precise point mutations were successfully introduced into three target sites in rice using base editing, hence providing an effective and efficient tool for targeted gene editing in plants [176]. The most precise and efficient version of Cas9 devoid of the nuclease activity (dCas9) is composed of Cas9 nickase (Cas9-D10A) and cytidine deaminase. This version leads to base transitions from G → A or C → T, along with uracil glycosylase inhibitor (UGI) inhibiting base excision repair. The improved version of Cas9 known as BE3 (APOBEC1-XTEN-nCas9-UGI) constitutes the third generation of base editor, BE3 which cleaves the non-complementary strand and causes specific base-editing via manipulating DNA repair machinery in cells. The base editing efficiency of BE3 ranges from 15–75 percent with minimum indel formation in case of animals [69]. Site-specific C to T changes can be produced in various plant species via codon-optimized base editors. The human APOBEC3A-based plant cytidine base editor was used in wheat, rice and potato to convert C to T, independently of the sequence context [175,177]. It has been found that deamination window of base editing in plants is wider than in animal cells with almost no indel formation in the edited plants. Consequently, base editing is highly specific and a potent tool in plants for generating point mutations to improve crop genetics and breeding.

Cytidine deaminase not only converts desirable C, but also converts other Cs within the deamination window, and has been recently engineered to reduce its window from ~5 to 1–2 bases [99]. However, CBEs generate more indels, off-target editing, and undesired mutations than do ABEs (164). Furthermore, the target range of base editing has been expanded by using several engineered and natural Cas9 variants with diverse PAM requirements. Hence, base editing widens the efficacy and scope of CRISPR-Cas9-mediated genome-editing for producing point mutations in plants, ultimately making it possible to target every single nucleotide within the genome.

Thus far, all the base editors described above mediate C:G → T:A conversion. However, seventh generation ABEs have been developed to change a targeted A to G and T to C in the opposite strand [178]. Ideally, adenosine deaminase fusion with the catalytically impaired CRISPR/Cas9 mutant would produce ABE. However, there was no enzyme identified to deaminate adenine in DNA [164]. A group of researchers conducted protein evolution and protein engineering to develop an adenine deaminase that can operate on a genomic DNA [178]. The evolution of tRNA adenosine deaminase (ecTadA) in *E. coli*, and the introduction of some mutations gave rise to TadA* which can deaminate efficiently about 53 percent adenine in DNA. Amongst four ABE classes reported, ABE7.10 is the most effective, and likes to target A at 4–7 protospacer positions, whereas the other three ABE6.3, ABE7.8 and ABE7.9 perform well when A is at position 8–10. These ABE’s catalyze A → I deamination, which is then recognized as G by the DNA polymerase, and after replication, the A–T base pair gets finally converted to a G–C one. This early development specifying the role of ABEs was reported in rabbit, mouse, wheat, rice, Arabidopsis, *Brassica napus*, and oilseed rape [172,173,177,179,180,181].

RNA base editing can offer great experiences for the life sciences and possibly for medicine. Due to the single-stranded nature of RNA, 12 promising base editors that function on RNA instead of six that operate on dsDNA are required to cover up all the possible variations. The RNA base editor (RBE) has been created by catalytically fusing inactive nucleases (dCas13b) with adenosine deaminases from ADAR (Adenosine Deaminase Acting on RNA) family. In RNA, RBEs convert a targeted base A to an I, and RNA base editing alters the protein with no permanent alterations in the genome. Recently, researchers have developed the REPAIR (RNA editing for programmable A to I replacement) system as a result of fusion between catalytically dead RNA guided Cas13 enzyme (dCas13) with ADAR2 deaminase [182,183]. As splicing and translation machinery reads inosine as guanosine, incorrect mutations of G → A could be then corrected by the REPAIR platform [184]. It was reported that REPAIRv1 virtually targets all RNA because Cas13 does not need PAM, whereas REPAIRv2 is an improved version because it decreases the off-target editing by 900-fold than REPAIRv1 [182].

Base editing has been used to regulate the RNA splicing pathways by generating point mutations at a splice donor site (GU) and a splice acceptor site (AG) at the 5′ and 3′ end, respectively, resulting in mis-splicing and loss of specific splice forms. In a recent study, point mutations at the splicing acceptor site of an intron by A → G conversion leading to changed splicing process of AtPDS mRNA [172]. 5′ splice donor sites have been disrupted by converting G to A using Cas9-directed base editor in four *Arabidopsis* genes resulting in intron retention of *AtHAB* [185]. Silencing of AS of HAB_1.1_ revealed its role in abscisic acid signaling, whereas manipulates the splicing of AS of RS_31_A and validated its function for the first time in plant response to genotoxic treatment. Another group of researchers have used Cas9-derived cytosine base editor to convert C → T by disrupting the highly conserved intron acceptor site AG or donor site GT, thereby creating mRNA mis-splicing-induced null mutants of *AtMTA* in *Arabidopsis* in T_2_ generation and double null mutant of *GL1-1* and *NAL1* genes in rice in T_0_ generation [186]. This type of manipulation of mRNA splicing by base editing in plants and other eukaryotes provides an efficient tool for validating the gene-splicing function and regulation.

The base editors could provide a ‘high-density mutant population’ and assist the artificial evolution of agronomically relevant loci, unlike the random mutagenesis-mediated TILLING technique, which creates the least mutation density for a particular gene of interest [187]. Broad-spectrum resistance in potato achieved by in vitro evolution of the NBS/LRR domain was found to be connected to plant fitness cost, which will possibly be removed by the random mutagenesis technique [188].

One of the most critical and demanding factors for the therapeutic achievement of BEs and Cas-derived gene-editing tools is the safe and efficient release of the editing reagents to the target cells [189]. The important factor for attaining successful delivery is selection of the suitable cargo [DNA/ RNA/RNP (ribonucleoprotein)], delivery vehicle (physical/chemical/viral) and size of the Bes [189,190]. Besides DNA-free base-editing approaches, the detection of new small size Cas9 orthologs, such as CasX, would assist the delivery of effective therapeutics [191]. Further advancements in modulating the BEs’ expression and overcoming the sequence preferences of CBEs would enhance their efficiency.

The target base and specific PAM sequence (NGG PAM for SpCas9) ought to be within a narrow base-editing window for successful base editing [13,164,178,191]. The limitation of this specific PAM requirement reduces the editing efficiency in plants. To expand the PAM compatibility and scope of base editing, many research groups have produced new ABE and CBE base editors using Cas9 variants, which recognize PAMs from the NGG motif [37,38,173,192,193].

Recently, Ren and coworkers demonstrated PAM-less plant genome editing in rice and the Dahurian larch, using an engineered SpRY Cas9 variant [194,195]. Using the non-homologous end joining (NHEJ) repair pathway, they have efficiently induced targeted mutagenesis at relaxed PAM sites in rice and protoplasts of Dahurian larch. In addition, a SpRY-based cytosine base editor has been developed and established by the directed evolution of new herbicide resistant *OsALS* alleles in rice. In the same way, an extremely active SpRY adenine base editor has been developed on the basis of ABE8e [196] and SpRY-ABE8e was capable of targeting relaxed PAM sites in rice plants, showing up to 79% editing efficiency with high product purity. Therefore, the SpRY toolbox breaches a PAM restriction barrier in the case of plant genome engineering by facilitating DNA editing in a PAM-less approach. With the advent of novel gene-editing tools based on SpRY, researchers could effortlessly perform high-resolution genome engineering, and can use SpCas9 for targeting sites carrying NGG PAMs, SpCas9-NG for NGH PAMs (where H is A, C, or T), ScCas9 for NHG PAMs, and SpRY for NAH PAMs. These improved base editors can boost the base-editing efficiency and increase its reach in targeting diverse sites in crop plants.

BEs have been widely exploited to install precise base changes, and the generation of undesired base changes (C → A or C → G), bystander and off-target edits, and indels notably impedes their use in therapeutics. However, such undesired edits are less challenging for crop improvement since desired edits and indels may be created on separate alleles, which could be fixed through segregation and assortment [164].

## 9. Prime Editing Systems for Precision Editing of Plant Genomes

Base editing in plants has overtaken HDR-mediated substitutions regarding engineering its efficiency and product purity. However, many mutations, such as base insertions and transversions, cannot be processed by existing base editors, which significantly limits the use of base-editing systems. Consequently, novel systems that confer a wide range of genome editing are greatly anticipated in plants. Recently, most versatile and precise prime editors (PEs) were produced to create mutations beyond DSBs or donor DNA [197]. A prime-editing guide RNA (pegRNA) was designed to direct site-specific nicking by nCas9 in order to install customizable mutations into target genomic locus. PEs could efficiently install all possible base conversions, deletions and insertions in a broader targeting range with limited by-products in mammalian cells, therefore expanding the molecular toolbox of genome editing [197].

In another study, a plant based prime editor 2 (pPE2) system has been designed to induce targeted mutation on a HPT-ATG reporter in rice [198]. The results demonstrated that the pPE2 system might induce programmable base transitions, base transversions, inversions at different genomic sites, specifying adaptability of this system in other plant species. They achieved 0–31.3 percent efficient editing frequency in T_0_ plants, indicating that the efficiency of pPE2 differed significantly at different genomic sites using pegRNAs of different structures. In order to improve editing efficiency of pPE2, gRNAs were incorporated into the pPE2 system ensuing the PE3 and PE3b approach in human cells. Conversely, pPE3 prime editing platform induced only similar or even lesser editing frequencies at the tested genomic sites. Besides, surrogate pPE2 system was developed by inserting the HPT-ATG reporter to supplement the prime-edited cells. Using this surrogate pPE2 system, DNA editing was effortlessly identified in the resistant calli transformed as a result of the greater screening efficiency of edited cells. To a large extent, the authors reported that such type of prime-editing systems might provide useful and flexible editing in rice genome.

Lin and coworkers used prime editors in cereal plants through optimizing promoter, codon and editing conditions [199]. The set of plant prime editors allows for point mutations, deletions and insertions in wheat and rice protoplasts. They selected six genes from each rice and wheat plant and constructed 21 pegRNAs to study the two-plant prime editor (PPE) systems i.e., PPE2 and PPE3 (or PPE3b). The PPE systems in rice induced 3-bp insertions at a frequency of 2.0 percent at *OsCDC48-T2,* 6-bp deletions at 8.2 percent frequency at *OsCDC48-T1* and the six different single nucleotide substitutions including G-to-T, C-to-T, G-to-A, A-to-G, C-to-A and T-to-A at 5.7% frequencies. Regenerated edited rice progeny was achieved at frequencies of up to 21.8%. In the case of wheat, the frequencies of single nucleotide substitutions, such as A-to-T, T-to-G, G-to-C, C-to-G and C-to-A, attained to 1.4%. In the plant protoplast systems, PPE3 and PPE3b had a similar editing efficiency, signifying the nicking of sgRNA does not necessarily increase prime editing efficiencies, in contrast with the observations recorded in mammalian cells [197], even though plant prime editors are less efficient for inducing transition point mutations than base editors. This investigation indicates that PPEs can produce transversions, insertions, substitutions, and deletions. The flexibility of prime editing in plants thus has the unprecedented power to improve both functional genomics and plant breeding research.

The major limiting factor of PE is its low editing efficiency. It has been reported that the editing efficiency of PE ranges from 0.03–21.8% in plant cells, i.e., much lower than that in human cells (20–50%) [198]. Secondly, prime editors possess a short editing window (i.e., size of RT template length), with 12–16 nt [197]. While 30–40 nt long editing windows have also been reported [199]. However, the success of using prime editors with longer editing window relies on the genomic sequence content of the target region, with a few target sites providing long editing windows, while others did not [199].

To outweigh the above mentioned major shortcomings of existing prime editors, researchers should focus on a multifaceted knowledge of the design principle of prime editing technology, streamline the parameters influencing the editing efficiency, and expand the editing window. Even though there are some approaches to designing prime editors for plant and animal systems [198,199], the design principle of prime editing has not been investigated comprehensively. The current proposal depends on the experimental data from the editing of a very narrow range of genomic loci (25 endogenous loci in plants and 12 endogenous loci in human cells), besides human cell lines, yeast cells, and the protoplast of rice and wheat [198,199]. Currently existing prime editors reported so far in plants can be employed to edit only one target site at a time. However, various traits are controlled by multiple genes or QTLs in the case of plants [200,201,202,203,204]. Furthermore, activation of any plant biosynthetic or metabolic pathway generally requires engineering multiple genes simultaneously. Thus, currently existing prime editing technology couldn’t be applied to edit multiple genes concurrently. Another technical limitation of prime editing is the size of the prime editing construct (~20 kb), which is fairly large, making it inefficient to transform into plants. The Cas9 orthologs smaller in size for example CasX [191] could decrease the size of the prime editor and allow the PE delivery into the plant cells.

## 10. Conclusions

The application of genome editing technologies in plants facilitates a wide range of opportunities for plant breeding. Genome editing leads to precise and targeted mutagenesis and forms the basis for many next-generations breeding tools that will certainly transform agriculture in the future. It becomes necessary to explore all the strategies in order to exploit the unprecedented potential of plant genome engineering. Genome editing can allow for a combination of different genetic traits for designing future crops. Moreover, such precision engineering, when employed for rapid plant breeding, results in products similar to that of conventional breeding. However, genome editing-based next-generation breeding will entirely replace classical breeding approaches; only when integrated with other technologies, such as genomic selection high-throughput sequencing and speed breeding, can we ensure the widespread applications of genome editing in agriculture. This multidisciplinary approach promises to advance plant breeding to increase the yield potential of food safety and security.

Winning Nobel Prize, the discovery of genome editing technology in the CRISPR systems has revolutionized the field of genetic engineering, especially plant genome editing, by providing plant genome editing technology with ease, quality, and precision. In fact, CRISPR/Cas9 is not just clippers for making DNA breaks anymore. This can change single target nucleotides without the requirement of a foreign DNA or DNA breakage, hence mimicking natural mutations, but with the trait of interest. The toolbox continues to be streamlined with newly designed CRISPR/Cas systems, effector modules and Cas proteins.

While base-editing technology evolved rapidly by fine-tuning the architecture of BEs to increase the efficiency, targetability, and purity of the edited product, there are many challenges that need to be overcome to utilize this technology to its full potential. Many BEs are available with nickase and dead-nuclease variants that facilitate more specific genomic editing, but they are not always as efficient as the original BEs developed with SpCas9 (D10A). Further evolution of Cas9 proteins and the discovery of new nucleases with more PAM plasticity would broaden the scope of genome targeting, while maintaining editing efficiency. For example, for editing T-rich genomic regions, a Cas12a-ABE could be developed, but does not yet exist.

New ground-breaking prime editing technology is an exciting method for more precision plant genome engineering that can put new genetic information directly into a designated gene locus, thus extending the genome editing outlook and capabilities to a greater extent. Prime editing is in its early stage of development. There are still some technical limitations that need to be addressed, and therefore more research needs to be conducted in order to optimize the system for plants. Here we have emphasized few key limitations of the technology and provided some implications on how we can improve it further. Regardless of various technical limitations and challenges, it is clear that prime editing will play a leading role in comparison to other genome-editing technologies for basic plant biology research and crop improvement in the near future.

Interestingly, researchers have already succeeded in knock-in fragments of DNA beyond donor DNA and the HDR pathway at the target site. This might be useful in the editing of plant genomes, since the knock-in gene is challenging in plant cells. Multiplex genome editing through CRISPR/Cas9 may be used to replicate the domestication process in a shorter time during evolution, with the involvement of the fast and suitable production of new plants with desirable and attractive traits. Simultaneous targeting of multiple sites can encourage deletions between target sites with defined sizes [5].

## Figures and Tables

**Table 1:** Comparative table of engineered nucleases.

S.No.	Properties	ZFN	TALEN	CRISPR-cas9	References
1	Target site	20–35bp	20–40bp	20–23bp	[24,25,26,27,28]
2	Nuclease	Two molecules of fokI	Two molecules of fokI	Cas9	
3	Efficiency	High	High	High	
4	Identification molecule	Protein-DNA	Protein-DNA	RNA-DNA hybrid	
5	Cost	High	Moderate	Low	
6	Limitation	Time consuming and laborious	Laborious	Off-targeting	

**Table 2:** DNA-free genome editing approaches used in recent studies.

S.No.	Plant Species	Trait	GE Technique	Tissue	Delivery System	Method	References
1 2 3 4	*Arabidopsis thaliana* *Lactuca sativa* *Nicotiana attenuate* *Oryza sativa*	POC	CRISPR/Cas9 RNPs	Protoplast	PEG fusion	Targeted deep sequencing	[117]
5	*Nicotianabenthamiana*	POC Herbicide resistance	TALEN		mRNA protoplasts PEG fusion	n.d	[120]
6	*Chlamydomonasreinhardtii*	Yield Abiotic stress	CRISPR/Cas 9 RNPs	Single cells	Electroporation	Deep sequencing	[121]
7	*Ch.reinhardtii*	POC	CRISPR/Cas9 RNPs	Single cells	Electroporation	WGS	[122]
8 9	*Malus domestica* *Vitis vinifera*	Biotic stress	CRISPR/Cas9 RNPs	Protoplasts	PEG-fusion	n.d	[123]
10	*Zea mays*	Male sterility Herbicide tolerance	CRISPR/Cas9 RNPs	Immature embryos	Biolistic	n.d	[57]
11	Petunia hybrid	Herbicide Resistance	CRISPR/Cas 9 RNPs	Protoplasts	PEG-fusion	n.d	[124]
12	*Triticumaestivum*	Yield	CRISPR/Cas 9	immature	Biolistic	PCR-RE	[112]
13	Non-albicans candida	POC	CRISPR/Cpf1 RNPs	Single cells	Electroporation	n.d	[115]
14	*Ch.reinhardtii*	Poc, Gene	CRISPR/Cpf1	Single cells	Electroporation	n.d	[125]
15 16	*Glycine max* *N. attenuate*	Fat synthesis POC	CRISPR/Cpf1 RNPs	Protoplasts	PEG- fusion	Targeted deep sequencing	[126,127]
17	*T. aestivum*	Yield	CRISPR/Cas 9 RNPs	Protoplasts, Immature embryos	PEG-fusion, Biolistic	Sanger sequencing	[128]
18	*Solanum tuberosum*	Starch synthesis	CRISPR/Cas9 RNPs	Protoplasts	PEG-fusion	n.d	[129]
19	*T. aestivum*	Yield/POC	CRISPR/Cas9	Protoplasts	PEG-fusion	n.d	[128]
20	*O. sativa*	Yield/POC	Cpf1 RNPs, TALENS proteins	Immature embryos	PEG-fusion	n.d	[128,130]

**Table 3:** CRISPR/Cas-mediated base editing in plants.

S.no.	Plant	Gene Targeted	Base Editor	Delivery	Editing Frequency	Indel Frequency	References
1	*Arabidopsis*	*ALS*	BE3	*Agrobaterium* mediated	1.7%	NR	[165]
2	Rice	*PDS*, two loci in *OsSBEIIB*	BE3	*Agrobaterium* mediated	0.1–20%	0–9.61	[63]
3	Rice	*NRT1.1B*, *SLR1*	BE3(-UGI)	*Agrobaterium* mediated	0–13.3%	10%	[166]
4	Rice	*CDC48*	BE3	*Agrobaterium* mediated	43.5%	0%	[167]
5	Maize	*CENH3*	BE3	*Agrobaterium* mediated	10%	NR	[167]
6	Wheat	*LOX2*	BE3	Particle bombardment	1.25%	0%	[167]
7	Rice	*ALS*, *FTIP1e*	Target-AID	*Agrobaterium* mediated	6–89%	10–62%	[168]
8	Tomato	*DELLA*, *ETR1*	Target-AID	*Agrobaterium* mediated	41–92%	16–69%	[168]
9	Rice	*CERK1*, *SERK1*, *SERK2*, *Ipa1*, *pita*, *BRI-1*	BE3	*Agrobaterium* mediated	0–38.9%	0%	[169]
10	Rice	ACC, ALS, CDC48, DEP1, NRT1.1B	ABE7.10	*Agrobaterium* mediated	3.2–59.1%	0%	[56]
11	Wheat	DEP1, GW2	ABE7.10	Particle bombardment	0.4–1.1%	0%	[170]
12	Rice calli	MPK6, MPK13, SERK2, WRKY45, Tms9-1	ABE7.10 ABE7.8	*Agrobaterium* mediated	0–62.26%	0%	[171]
13	*Arabidopsis*	FT, PDS3	ABE7.10	*Agrobaterium* mediated	0–85%	NR	[172]
14	*Brassica napus*	ALS, PDS	ABE7.10 ABE6.3, ABE7.8, ABE7.9	Protoplast transformation	8.8%	<0.1%	[172]
15	Rice	SPL14, SLR1, SPL16, SPL18 SPL14, SPL17 SPL16, SPL18	ABE7.18 ABE-Sa Sa Cas9-nickase	*Agrobaterium* mediated	12.5–26% 17–61%	0%	[173]
16	Rice	SPL13, SPL14, SPL16, SPL17 SPL18, GRF4 TOE1, IDS1 MTN1, SNB PMS1, PMS3 SNB	ABE-Sa ABE-VQR ABE-VRER ABE-SaKKH BE3, VQR-BE3 SaKKH-BE3	*Agrobaterium* mediated Particle bombardment	0–74.3% 0–80% 0%	NR	[174]
17	Wheat	ALS, MTL	hA3A-BE	Particle bombardment	16.7–22.5%	0%	[175]
18	Rice	CDC48	hA3A-BE NRT1.1B	*Agrobaterium* mediated	44–83%	0%	[175]
19	Potato	GBSS	hA3A-BE	Protoplast transformation	6.5%	0%	[175]
20	Rice calli	EPSPS, ALS, DL	Target-AID-NG	*Agrobaterium* mediated	5–95.5%	0–68%	[38]

## Data Availability

Not applicable.
